# Culturing Toxic Benthic Blooms: The Fate of Natural Biofilms in a Microcosm System

**DOI:** 10.3390/microorganisms5030046

**Published:** 2017-08-06

**Authors:** Francesca Di Pippo, Roberta Congestri

**Affiliations:** 1 CNR-IRSA, National Research Council, Water Research Institute, Area della Ricerca di Roma 1, Monterotondo Stazione, Rome 00015, Italy; dipippo@irsa.cnr.it; 2 CNR–IAMC, National Research Council, Institute for Coastal Marine Environment, Località Sa Mardini, Torregrande, Oristano 09170, Italy; 3 LBA-Laboratory for Biology of Algae, Department of Biology, University of Rome “Tor Vergata”, Rome 00133, Italy

**Keywords:** benthic harmful algal blooms (BHABs), benthic dinoflagellates, *Ostreopsis* cf. *ovata*, biofilm cultures, confocal microscopy

## Abstract

A microcosm designed for culturing aquatic phototrophic biofilms on artificial substrata was used to perform experiments with microphytobenthos sampled during summer toxic outbreaks of *Ostreopsis* cf. *ovata* along the Middle Tyrrhenian coast. This dynamic approach aimed at exploring the unique and complex nature of *O.* cf. *ovata* bloom development in the benthic system. Epibenthic assemblages were used as inocula for co-cultures of bloom organisms on polycarbonate slides at controlled environmental conditions. Biofilm surface adhesion, growth, and spatial structure were evaluated along with shifts in composition and matrix production in a low disturbance regime, simulating source habitat. Initial adhesion and substratum colonisation appeared as stochastic processes, then community structure and physiognomy markedly changed with time. Dominance of filamentous cyanobacteria and diatoms, and dense clusters of *Amphidinium* cf. *carterae* at the mature biofilm phases, were recorded by light and confocal microscopy, whilst *O.* cf. *ovata* growth was visibly limited in the late culture phases. Life-form strategies, competitiveness for resources, and possibly allelopathic interactions shaped biofilm structure during culture growth. HPLC (High Performance Liquid Chromatography) analysis of exopolysaccharidic matrix revealed variations in sugar total amounts and composition. No toxic compounds were detected in the final communities tested by LC-MS (Liquid Chromatography- Mass Spectrometry) and MALDI-TOF MS (Matrix Assisted Laser Desorption Ionization Time OF Flight Mass Spectroscopy) techniques.

## 1. Introduction

*Ostreopsis* cf. *ovata*, a toxic benthic dinoflagellate, has considerably expanded its distribution in temperate areas in the last 20 years, forming recurrent benthic harmful algal blooms (BHABs) in the Mediterranean basin [1,2]. BHABs rapidly spread along Italian Middle Tyrrhenian coast, since their first record in 1999 [3,4], with summer outbreaks sustained by the most abundant and so far widely distributed *Ostreopsis* Mediterranean species [5,6,7,8]. On these occasions, BHABs come in brownish, mucous cell aggregates, visible to the naked eye, proliferating on biotic and abiotic substrata of relatively shallow and sheltered coastal stretches favored by low water motion and increased input of organic nutrients [9,10,11]. Complex and diverse microphytobenthic associations co-occur with *O.* cf. *ovata* blooming cells, especially diatoms and cyanobacteria, all enmeshed in a common exopolymeric matrix that probably plays a central role in cell attachment to surfaces, colony formation, and bloom “nutrition” and dispersion [7,10,12,13,14]. Undesirable impact of *Ostreopsis* BHABs includes production of palytoxin-like compounds that caused benthic biocenosis sufferings and mass mortalities, along with human intoxications and also severe economic impact [15,16,17,18]. A global increase in BHABs in temperate and tropical regions has been recorded, possibly due to more effective monitoring and also to anthropogenic factors and global climate change [1,19]. In any case, it is still a matter of debate whether global warming will increase *Ostreopsis* BHABs distribution, periodicity, and intensity, as the physiological response to temperature and pH in culture and field studies did not infer a clear effect of these variations on BHABs [1].

Much effort has been put in the monitoring of *O.* cf. *ovata* presence and blooms along the Italian coast and numerous studies focused on *Ostreopsis* phylogeography, taxonomy, ultrastructure, toxicology, and life-cycle with in depth understanding of the growth and toxicity responses to environmental conditions of several isolates in batch cultures [9,10,11,12,14,20,21,22].

Despite these considerable scientific advances, the unique and complex nature of the BHABs still poses questions as researchers’ acquaintance of bloom events mostly focuses on the planktonic system. Current knowledge of what affects BHAB organism presence, bloom onset, deleterious impact, and demise in benthic habitats is still scant, also due to the interplay between triggering factors (temperature, salinity, irradiance, microalgae nutrition and interactions) in bloom development, shifts, and dispersal in nature.

The use of microcosms to culture benthic communities on artificial substrata can represent a more realistic and dynamic approach to understand benthic bloom processes, starting from initial cell recruitment and adhesion to surfaces, to their growth in multilayered/network-like mature communities, their eventual detachment from host substrata and dispersion in the water column [23,24,25]. In this scenario, an incubator prototype specially designed for culturing aquatic phototrophic biofilms [26] was used to grow the whole microphytobenthic communities sampled during toxic *O.* cf. *ovata* outbreaks on artificial substrata at controlled environmental conditions. A microcosm approach provided the possibility to co-culture bloom microorganisms on polycarbonate slides in a moving film of medium in order to minimize planktonic growth under controlled temperature and light conditions in closed photobioreactors. 

BHAB communities were sampled from bryozoans and macroalgal thalli at two “hot spots”, along the Latium coast (Middle Tyrrhenian Sea) identified during decadal monitoring of *O.* cf. *ovata* outbreak periodicity and intensity. Epibenthic biofilm samples were inoculated in the flow lane microcosm and cell growth and biofilm development were followed by means of automatic, real-time monitoring of biomass accumulation. This also allowed depiction of interaction processes of the bloom community with artificial substrata as cell accrual, surface adhesion, and coverage in confocal laser scanning microscopy of intact biofilms. A combination of confocal laser scanning microscope (CLSM) imaging and biochemical analysis was also applied to evidence spatial/biochemical interactions between biofilm members, compositional shifts, and matrix and toxin production in a low disturbance regime, simulating the conditions that support bloom development and persistence in nature.

## 2. Materials and Methods 

### 2.1. Study Area and Sampling 

Sampling was conducted in July and August 2009 at two stations along the Latium coast, Italy, Middle Tyrrhenian Sea (Formia, Site 1, 41°15′00′′ N, 13°37′00′′ E; and Sperlonga, Site 2, 41°15′32′′ N, 13°25′58′′ E), recurrently affected by *Ostreospis* cf. *ovata* summer blooms [3].

Both sites are shallow (mean depth 1.5 m), semi-enclosed rocky locations with low hydrodynamism; surface temperature, dissolved oxygen, salinity, and pH values were registered by YSI probes (YSI Inc., Yellow Springs, Oh, USA, Table 1). Bryozoan samples, in July, and macroalgal thalli (*Cystoseira* sp., Phaeophyceae), in August, visibly colonised by conspicuous brownish mucous material, were opportunely collected in three replicates at 0.3 m depth, following the protocol reported in [27]. Briefly, the host organisms were hooded in plastic bags and promptly closed underwater to avoid epibenthic cell loss, then transferred in plastic jars in the dark and refrigerated until laboratory analysis and preparation of culture inocula on the same day. 

### 2.2. Laboratory Analysis

#### 2.2.1. Preparation of Inocula

Host samples were shaken in the storage water to allow detachment of epibenthic cells, then rinsed with filtered seawater (WhatmanGF/C filters GE Healthcare, Little Chalfont, UK) to completely remove biofilm material. Rinsing suspensions containing biofilm cells were sieved (100 µm mesh) to remove larger organisms and detritus. Bryozoans and macroalgae were weighted to determine fresh and dry weight (FW, DW). 

#### 2.2.2. Counting of *Ostreopsis* cf. *ovata* Cells

Aliquots of biofilm suspensions after homogenization were fixed in Lugol’s iodine solution and left to settle for 24 h in 25 mL counting chambers. *Ostreopsis* cells were counted according to the Utermöhl method [28] using a Zeiss Axiovert 100 microscope. Cell concentrations were expressed as cells g^−1^ FW. 

### 2.3. Microcosm Cultures

2 L fresh biofilm suspensions were poured into 4 L bottles containing 2 L of K/2 medium [29] to obtain a 4 L culture inoculum for each suspension. Three growth experiments, named Run 1, Run 2, and Run 3 were performed with the three different inocula at the same environmental conditions (Table 1). 

**Figure 1:**
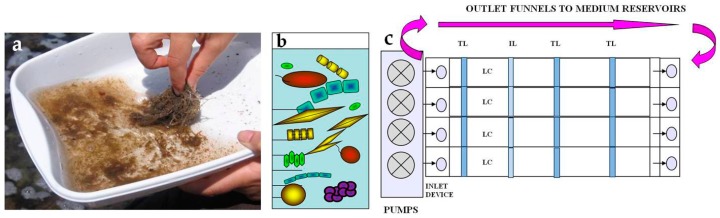
Methodological approach. Host organisms were sampled (**a**) and the biofilms washed off and collected (**b**). Cell suspensions were filtered and inoculated in the flow lane incubator (**c**).

The methodological approach is shown in Figure 1.

Two identical microcosms (biofilm growth systems) were used for July parallel cultures (Run 1 and Run 2) and one for the August experiment (Run 3), with simultaneous control of irradiance, temperature, and flow velocity. Each microcosm (UFZ Centre for Environmental Research, Magdeburg, Germany) contained four separate flow lanes (Figure 1) with integrated inlet and outlet devices at the start and the end of each lane. Each chamber had a 798 cm^2^ area made up of 42 removable polycarbonate slides, utilised as growth substrata. The inoculum was pumped from the 4 L reservoir through the inlet device within each lane with a laminar flow of 50 L h^−1^ over the growth surface. A turbulence reducer and a water temperature sensor were located at the inlet device of each chamber and the flow rate was valve-regulated. Temperature was set at 25 °C and irradiance at 110 μmol photons m^−2^·s^−1^. Fluorescent lamps (True-light 36 W AURALIGHT) were used in a 14/10 light/dark regime. The biofilm growth was followed for 50 days bloom duration at sampling sites, and accurately tracked on-line by the reduction in light transmittance measured in each chamber by nine light sensors positioned under the growth surface. Biofilm samples were collected at three temporal developmental phases: initial adhesion, active growth, and mature phase (corresponding to 90%, 50%, and 10% of the transmitted light, respectively) [26]. Each growth experiment ended when the recorded transmitted light was less than 10%, indicating the achievement of a stationary growth phase. Transmittance values were converted into a percentage of light absorbed by biofilms to obtain the growth curves [25,26]. The medium was changed at regular intervals (twice per week).

Biofilm Analyses:

- **Biodiversity**
  To assess biodiversity of phototrophs in natural and cultured communities, samples scraped from randomly selected slides were fixed with formaldehyde at 2% and glutaraldehyde at 2.5% final concentrations and then stored at 4 °C. Fixed samples were examined using a ZEISS Axioskop light microscope equipped with differential interference contrast (DIC) at 40× and 100× objectives. Samples were also observed at light microscope after staining for 10 min with Alcian Blue (AB) 1% in HCl 0.5 N (pH 0.5) or in 3% acetic acid (pH 2.5) to evaluate the presence of sulfated and carboxylic polysaccharides in the extracellular matrix [30].
- **Biomass**
  Cultured communities sampled at the three phases of development were scraped off the polycarbonate slides to evaluate the biomass. Biofilm biomass was determined as dry weight (DW) by oven drying samples at 60 °C for 72 h. Phototrophic biomass was assessed by determining Chl *a* concentration, extracted overnight in 90% acetone in the dark and then quantified spectrophotometrically according to [31].
- **Structure**
  The biofilm architecture and spatial distribution of phototrophs during the different phases of development was obtained by observing intact biofilm, after non-destructive, non-invasive sampling, at the confocal laser scanning microscope (CLSM) (Olympus FV1000, IX81 using Plan-Apochromatic 60× (NA 1.42, oil) objectives) at the Centre of Advanced Microscopy “P. Albertano”, Department of Biology, University of Rome “Tor Vergata”, Rome, Italy. CLSM was used in a multichannel mode, whereby the different channels mapped individual biofilm components. The excitation wavelengths were in the blue (488 nm, Ar), green (543 nm, Ar/HeNe), and red (636 nm, Ar/HeNe) regions. Data consisted of a set of two dimensional (2D), cross-sectional images in the *x*-*y* plane that were captured along the *z*-axis. The three-dimensional images were obtained through acquisitions in the XY plane, Z step size 0.5 µm, using the software package IMARIS 6.2.0 software (Bitplane AG, Zurich, Switzerland).
- **Spectral analyses**
  Spectral analysis (CLSM-SA) of different autofluorescence signals was also carried out on biofilm samples by using the *lambda scan* (*λ scan*) function of CLSM exciting specific regions of interest (1 µm^2^ ROIs) [32]. Lasers with the following excitation wavelengths: 488 nm; Ar, 543 nm; Ar/HeNe and red 635 nm; Ar/HeNe were used. Spectra were obtained for emission wavelengths ranging between 400 and 800 nm.
- **Matrix exopolysaccharides**
  Bound/capsular exopolysaccharides (CPS) were also extracted from mature communities using 0.1 M H_2_SO_4_ at 95 °C [33,34]. The carbohydrate fractions were measured spectrophotometrically using the phenol-sulphuric acid method [35]. In order to check the extraction efficiency of each eluent, the pellet obtained after the extraction was stained with Alcian Blue dyes, specific for acidic polysaccharides, to check localization of positive reaction in light microscopy [23]. CPS extracts were then analysed for their monosaccharide composition using RP-HPLC (reverse phase), using a Beckman Ultrasphere ODS (Octa Decyl Silane) column as reported in [36].
- **Toxins**
  *Ostreopsis* toxins were analysed in inocula and culture material by LC-MS (Orbitrap XL).
  MALDI-TOF mass spectrometry for cyanobacterial toxins was performed by AnagnosTec GmBH (Postdam, Germany) on mature biofilm samples.

## 3. Results and Discussion

The dynamic approach employed here represents the first attempt to culture the whole *O.* cf. *ovata* BHAB community on substrata to better understand microphytobenthos/biofilm development and shifts under simplified, stable environmental conditions that can mimic bloom evolution in sheltered, temperate waters during summer.

### 3.1. Epibenthic Communities

#### 3.1.1. Taxonomic Composition

**Figure 2:**
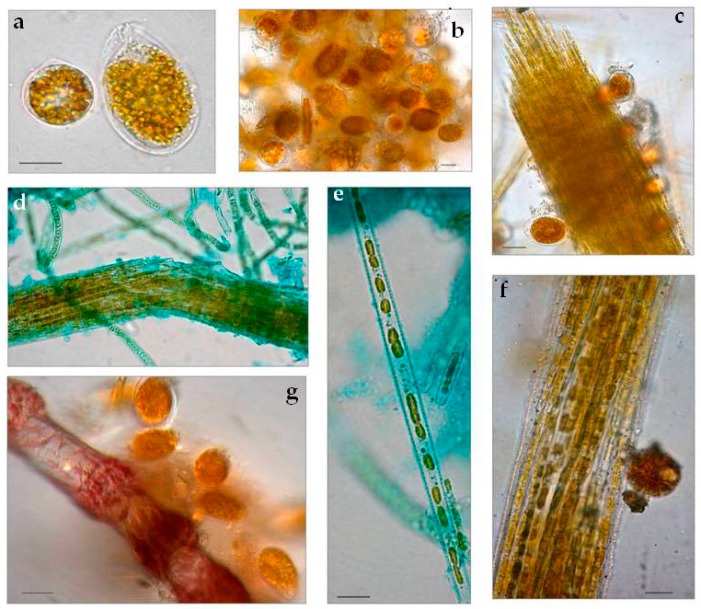
Light micrographs of July bloom communities washed off briozoa, *Ostreopsis* cf. *ovata,* and *Coolia monotis* (**a**), *O.* cf. *ovata* and centric diatoms (**b**), fascicle of *Nitzschia* cf. *martiana* cells bearing individuals of *O.* cf. *ovata* (**c**) and *C. monotis* (**f**). Alcian blue pH 2.5 staining: the exopolysaccharidic tube of *N.* cf. *martiana* and the sheaths of filamentous cyanobacteria are highlighted in (**d**) *N.* cf. *martiana* in valvar view (**e**), *O.* cf. *ovata* embedded in mucilage around an epizoic red alga branch (**g**).

Bryozoans and *Cystoseira* sp. (Phaeophyceae) hosted microphytobenthic communities with similar gross aspect and taxonomic composition irrespective of time and site of sampling. Epizoic and epiphytic associations macroscopically appeared as brownish mucous material, slightly diffluent and more or less adherent to the colonised surfaces. Light microscopy of host fragments revealed complex networks of microalgae and cyanobacteria. Epizoic associations in July samples included the dinoflagellates *Ostreopsis* cf. *ovata*, *Amphidinium* cf. *carterae*, *Coolia monotis,* and *Prorocentrum lima*. Among diatoms, solitary naviculoids and nitzschioid forms, along with tube dwelling and colonial species prevailed (*Amphora* spp., *Ceratoneis closterium, Entomoneis* sp., *Grammatophora* sp., *Lichmophora* spp., *Rhabdonema* sp.) over large centrics of *Coscinodiscus* and *Thalassiosira* genera. Fascicles of *Nitzschia* cf. *martiana* abundantly colonised bryozoans and had clusters of *O.* cf. *ovata* cells embedded in the mucopolysaccharidic material forming the diatom crenulated tube, as clearly evidenced by cytochemical staining in light microscopy (Figure 2). Oscillatorialean cyanobacteria, mostly less than 3 μm in diameter, and few coccal morphotypes were also largely present. *Cystoseira* sp. thalli, sampled in August, appeared to host proportionally more abundant *Ostreopsis* cf. *ovata* and *Coolia monotis* cells.

#### 3.1.2. *O*. cf. *ovata* Abundance

Inverted microscope counting showed abundances of 0.013, 0.062, and 0.103 cell g^−1^ FW for Run 1, Run 2, and Run 3 inocula used for the culture experiments in the microcosms. 

Recurrence of these composition patterns was also recorded during BHAB monitoring along Northern Adriatic sea coast [7] and along Catalonia coast (Spain, NW Mediterrenean sea) [37] and concentration data reflect ranges and variation observed in the Mediterranean basin [19].

### 3.2. Growth in the Microcosm

**Figure 3:**
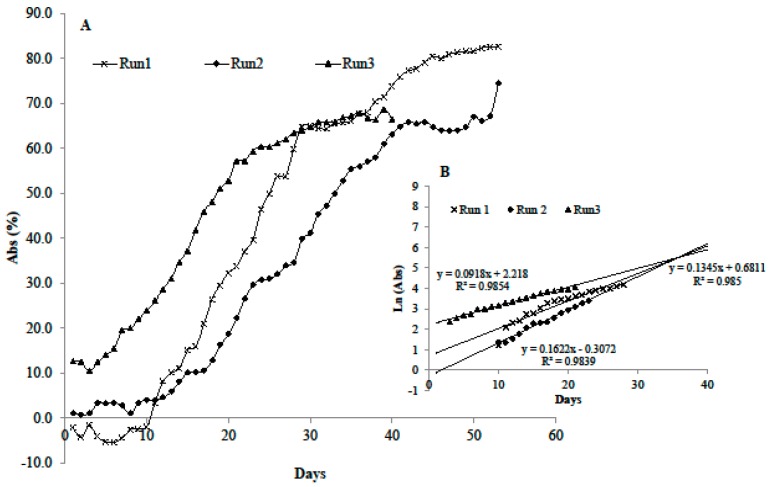
Growth curves obtained by transforming the on-line recorded light transmitted values in light absorbance (**A**). Linear portion of the natural log transformed data (**B**).

Biofilm did grow on the artificial substrata placed on the bottom of the microcosm lanes under high temperature and low disturbance simulating conditions that support bloom development in the sampling sites. Non-destructive, real-time monitoring of biomass accumulation was obtained by recordings of the subsurface light sensors. After inoculation, during which microorganisms settled and adhered onto the slides, a lag phase followed (Figure 3). Initial phases lasted only three days in Run 3 but up to ten in Run 1 and Run 2 cultures. Exponential growth (active phase) followed in the three growth experiments and cultures displayed different maximum growth rates, the highest registered for Run 1 and Run 2 communities. After a period of exponential increase, curves assumed a S-shape and reached a stationary phase indicating similar trends in biomass accrual. These trends are comparable to those obtained for marine biofilms of the Schelde estuary mudflats (The Netherlands), cultivated in the same system [24]. During the late development phase, biofilm sloughing occurred in Run 3 at day 38, likely due to a lower adhesive potential of the biofilm, resulting from lower exopolysaccharide content estimates in Run 3 cultures (0.012 mg cm^−2^) compared to Run 1 (0.038 mg cm^−2^) and Run 2 (0.028 mg cm^−2^). In any case, partial detachment of biofilm flocs was recorded during the experimental time in the three Runs, likely due to diffusion limitation caused by biofilm thickness. This can be considered one factor intrinsic to the biofilm causing dispersion of the BHAB cells in the water column in nature. 

### 3.3. Biofilm Observations

**Figure 4:**
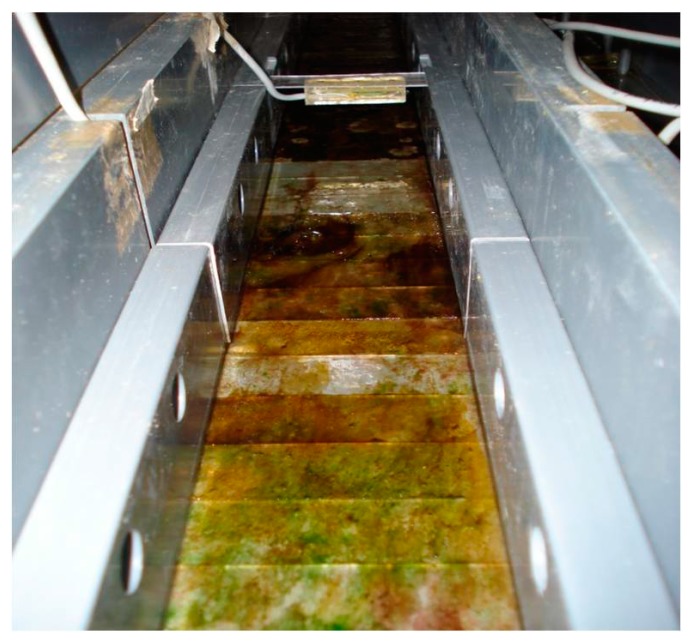
Microcosm lane with bottom slides colonised by biofilm. Coverage is patchy and differently pigmented portions are visible.

Macroscopic observations of biofilms were performed daily to monitor biofilm development on the slides. Initial adhesion and substratum colonisation appeared as stochastic processes in the three experiments performed. Cultures appeared as series of large patches, different in pigmentation that became more coherent with experimental time, when a more homogeneous coverage of substrata was visible. At the end of the experiments, corresponding to the late phase of development, biofilms were approximately 1 mm thick. At this phase, microorganisms formed macroscopic, mucilaginous, bright blue-green mats, showing a coherent structure, where the filamentous forms were more or less oriented to the flow direction of the culture medium (Figure 4).

**Figure 5:**
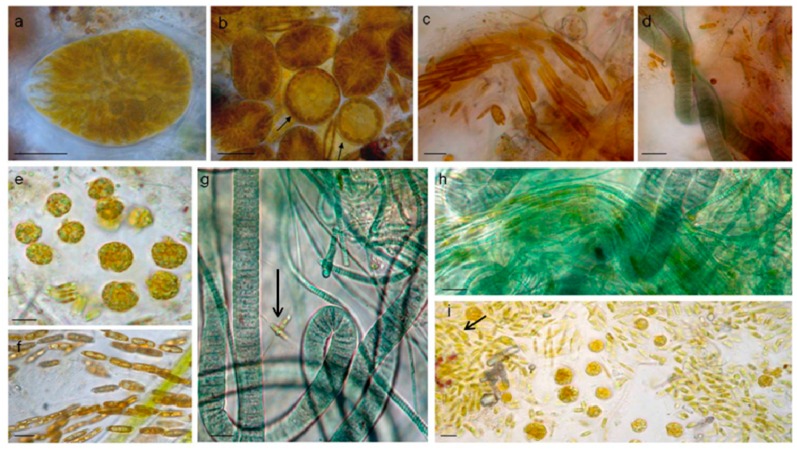
Light microscopy showed shifts in biofilm composition in all Runs and prevalence of cyanobacteria in late growth phases. *O.* cf. *ovata* cells were healthy and proliferating at initial (**a**,**b**) and active phases. *Coscinodiscus* sp. (**b, arrow**) at initial phase of Run 3. Tube dwelling diatoms (**c**,**f**); active phase, Run 2) were abundant in all biofilms, along with *Ceratoneis closterium* (**g, arrow**) and *Amphora* spp. layered cells (**i, arrow**), while Oscillatorialean cyanobacteria (**d**,**g**,**h**) dominated the last development phases. *Amphidinium* cf. *carterae* (**e**,**i**) was also abundant in the last phases. Bars = 10 μm.

Light microscopy observations of biofilm-fixed fragments scraped from randomly selected polycarbonate slides during the three Runs performed, showed a general reduction in taxon richness of cultures over experimental time. *O*. cf. *ovata* cells were healthy and proliferated at initial and active phases (Figure 5a,b), but then *Amphidinium* cf. *carterae* dominated among dinoflagellates and a general prevalence of filamentous, straight, or tightly coiled, non heterocytous cyanobacteria (*Leptolyngbya*, *Lyngbya*, *Oscillatoria*, *Microcoleous*, *Phormidium*, *Pseudanabaena*, *Spirulina* spp.) was observed in later phases (Figure 5 c–i). Among diatoms, tube-dwelling species dominated in late growth phases (*Parlibellus* sp.) along with the solitary *Amphora* spp. and *Ceratoneis closterium* (Figure 5c,g,i). This loss in diversity and shift to cyanobacterial dominance has also been reported in other experiments performed on phototrophic biofilms cultivated in photobioreactors [24,25].

### 3.4. Biofilm Matrix

**Figure 6:**
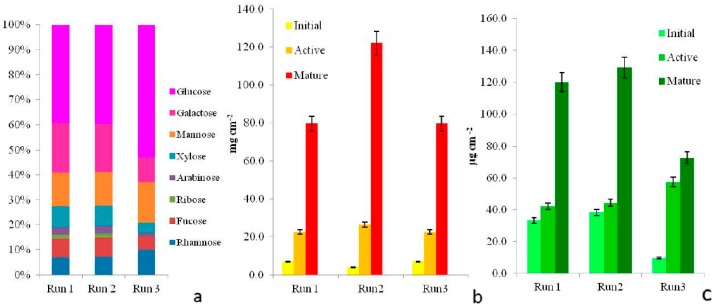
Biofilm capsular exopolysaccharides (CPS) monosaccharides in mature samples grown at each Run. Different proportions of 8 neutral sugars differed between Runs, data are expressed as mol % (**a**). Dry weight (**b**) and chlorophyll *a* content (**c**) values of biofilms at the three development phases, increasing trends over time were detected for both biomass indicators.

Biofilm microorganisms were immersed in an acidic exopolymeric matrix as revealed by light microscopy observations after biofilm staining with Alcian Blue dyes. Moreover, sulphated and carboxylated groups were present in the capsular polysaccharides of the cyanobacterial envelopes and in the tubes or diffluent mucilage produced by raphid diatoms. CPS extracted from all biofilm communities also contained at least seven different neutral sugars (Figure 6a). Findings on sugar composition in mature phase samples agreed with data obtained for cultured biofilms dominated by cyanobacteria [23,36]. Exopolysaccharide-producing microorganisms are favoured in nutrient supply and internal recycling, and this can explain their prevalence in the late development phase. EPS (Exopolymeric Substances) also provided high light screening of the outermost biofilm phototrophs, pointing to a key role of EPS production capacity in shaping the mature biofilm phase.

### 3.5. Biomass 

Due to the heterogeneous distribution of microorganisms on substrata and the high biomass development at the late growth phases, sampling for counting biofilm members was not considered as representative of the diversity observed. Thus, biomass accumulation was estimated as dry weight (mg cm^−2^) and chlorophyll *a* content (µg cm^−2^), confirming trends of growth curves (Figure 6b,c, respectively). 

### 3.6. Biofilm Structure

Confocal microscopy analyses were carried out by directly observing biofilms on the polycarbonate slides. This allowed preservation of biofilm integrity during sampling and analysis of the spatial relationships among biofilm members and with the substrata throughout biofilm development. Visualization of biofilm architecture, based on the autofluorescence signals of the different photosynthetic organisms, also allowed analysis of the inner components immersed in the biofilm thickness at the three sampled phases. 

**Figure 7:**
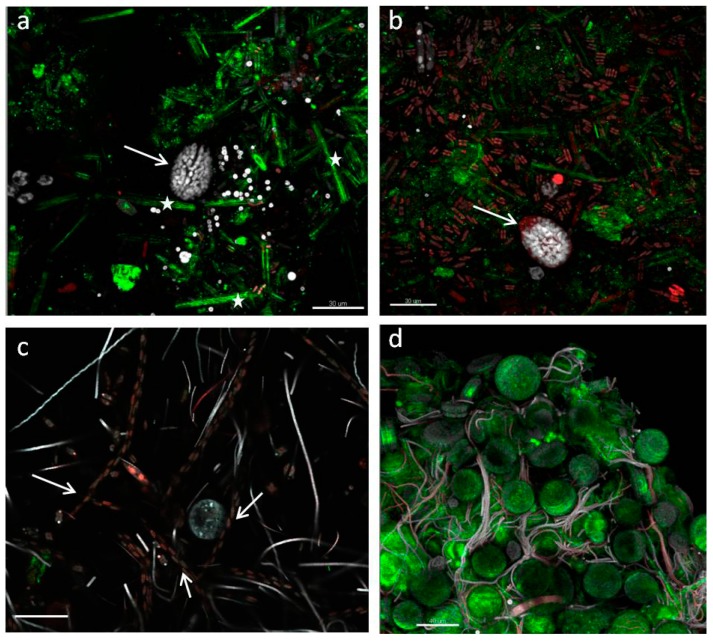
Confocal laser scanning microscopy (CLSM) 3D biofilm reconstruction at initial (**a**,**b**), and active (**c**,**d**) phases. *O.* cf. *ovata* cells are clearly visible in the median part of the uppermost images (**a,b, arrows**) showing numerous, elongated chloroplasts located at the cell periphery. Green signal was obtained with a 488-nm laser source, emission of frustule autofluorescence [39,40] is visible in (**a, asterisk**) where elongated diatoms occurr sparse on the slide, while a dense cluster of *Coscinodiscus* sp. lies interspersed with thin cyanobacterial trichomes in (**d**). White and red signals were captured after 543- and 635-nm laser excitation of accessory pigments and chlorophyll *a* of biofilm phototrophs. Filaments of small naviculoids are visible in pale red in (**c, arrows**) Bars = 30 (**a,b**), 40 (**c,d**) µm.

**Figure 8:**
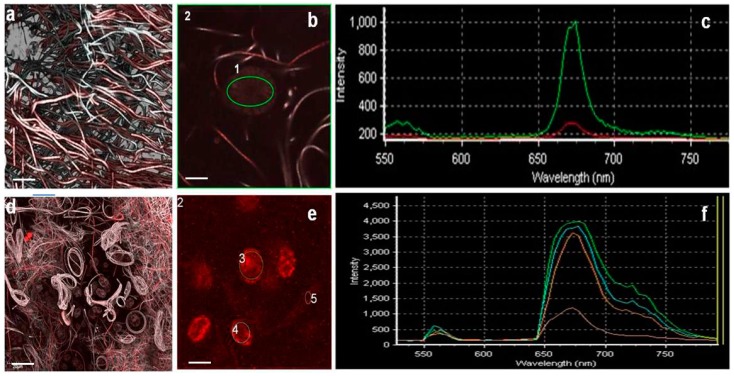
Spectral analysis at CLSM of *O.* cf. *ovata*, above, and *A.* cf. *carterae* after excitation with a 543 HeNe laser source. In (**a**,**d**) the original 3D reconstructions used for the SA, in (**b**,**e**), respectively, magnifications of the slices selected. Different regions of interest (ROIs) were acquired and labelled: 1 (encircling an *O.* cf. *ovata* cell) and 2 (encircling a substratum portion) of (**b**); 2 (encircling the growth substratum) and 3, 4, 5 (encircling *Amphidinium* cf. *carterae* cells) in (**e**). After excitation, the emission spectra relative to the selected ROIs where plotted (**c,f**), peaks in the 680-nm region are typical of peridinin–chlorophyll *a*-protein complexes present in both dinoflagellates. Bars = 30 µm.

An in depth observation of the “patchwork” coverage of initial phases revealed that 10-day-old biofilms already had a complex structure and thickness that could reach 150 μm in Run 1 (Figure 7a,b). *O.* cf. *ovata* cells were healthy and proliferating at initial and active biofilm phases. Spectral analysis of cells confirmed the results (Figure 8). Different regions of interest (ROI) were acquired and excited with different laser wavelength and the reported obtained spectra (Figure 8) showed active peridinin–chlorophyll *a*-protein complexes [38] for light absorption (Figure 8). The possibility to perform the spectral analysis of individual cells allowed discrimination between the different autofluorescent signals, providing a community fingerprint based on the properties of photosynthetic pigments.

**Figure 9:**
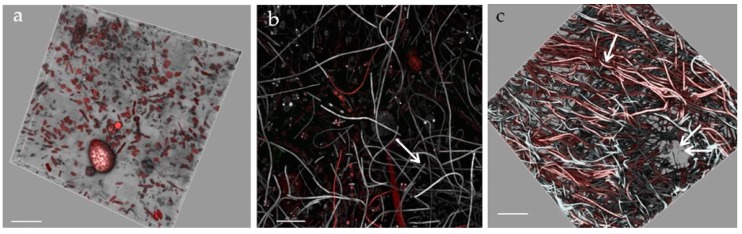
CLSM volume rendering of biofilm at initial phase (**a**), 3D reconstruction (**b**) and volume rendering (**c**) of samples at the active phase, signals are colour-coded as in Figure 7. Network (**b, arrow**) and layering (**c, arrow**) across the biofilm thickness are visible in the most advanced growth phases as well as the presence of voids (**c, double arrow**). Bars = 40 µm.

**Figure 10:**
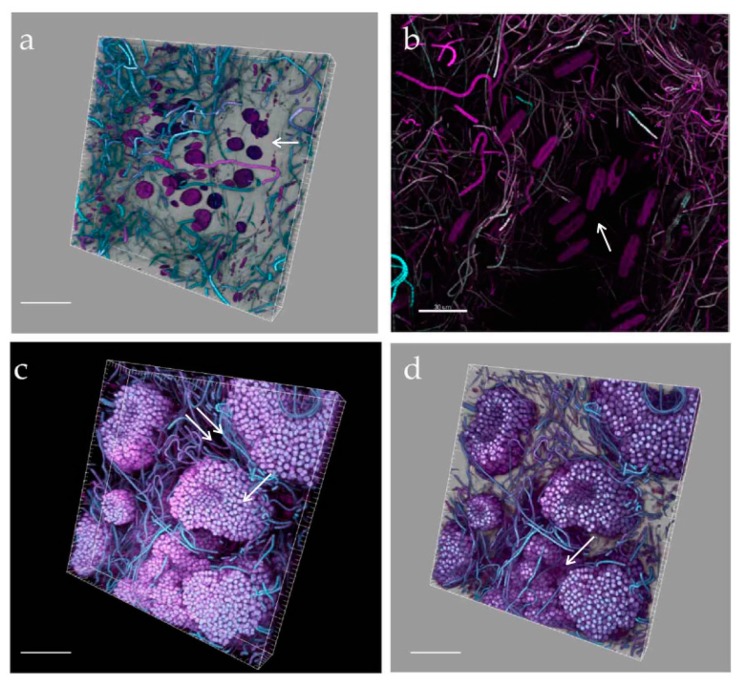
CLSM volume renderings (**a**,**c**,**d**), and 3D reconstruction (**b**) of biofilm at mature phases. Networks of diverse, more or less oriented, filamentous cyanobacteria lie close to clusters of *Amphidinium* cf. *carterae* cells, apparently excluding other biofilm organisms (**a, arrow**), and of large raphid diatoms (**b**, **arrow**). 3D renderings evidencing microcolonies of actively dividing coccal, colonial cyanobacteria (**c arrow**), forming channels (**c, double arrow**) and valleys (**d, arrow**)). White signal was obtained with a 488-nm laser source, magenta and cyan colour codes using 543-nm and 635-nm laser sources, respectively. Bars = 40 µm.

As the biofilm matured, so did its complexity with stratification and heterogeneous distribution of the organisms on substrata, resulting in the formation of voids and “valleys” (Figure 9 and Figure 10). Biofilm filamentous members, cyanobacteria and tube-dwelling diatoms, were mostly responsible of the layered structures observed (Figure 9c), while voids appeared especially around *Amphidinium* cf. *carterae* cell clusters, indicating exclusion of other biofilm members possibly due to the production of allelopathic compounds or mucous capsules around cell clusters (Figure 9c and Figure 10a) [23,41]. The ‘valley’ aspect was due to globular microcolonies of coccal cyanobacteria, evidencing active, multiple cell divisions (Figure 10c,d).

**Figure 11:**
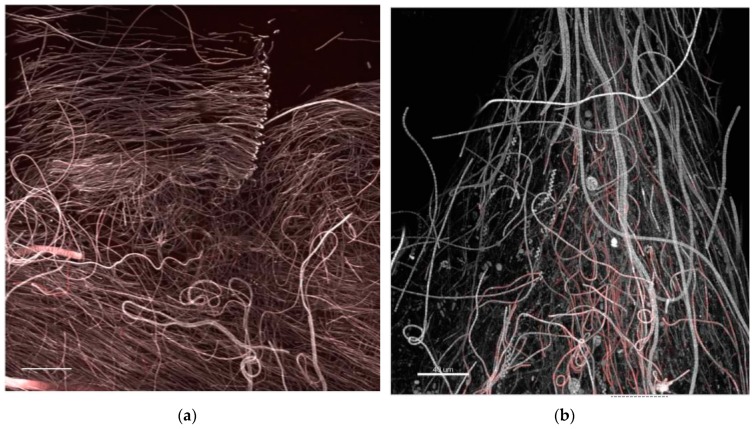
CLSM 3D recontructions of biofilm at mature phases showing layering of oriented, filamentous cyanobacteria (brighter autofluorescence at filament apex is possibly due to a light stimulus response) in (**a**) and occasional observation of loosely attached filamentous phototrophs or streamers, floating in flow direction (**b**), colour coded as in Figure 7. Bars = 40 µm.

Low-magnification observations carried out to evaluate the structure and proportions of the different phototrophic components at active and mature communities showed biofilms strongly characterized by the dominance of cyanobacteria and the abundant presence of only a few diatoms taxa (Figure 9a and Figure 10a,b,c). Cyanobacteria represented the best competitors in the advanced phases of community development probably due to their capacity to thrive in the low light microenvironments established across the biofilm thickness as also shown in [36]. This could imply a risk of massive cyanobacterial development in benthic habitats and toxicity outbreaks, although our simplified conditions excluded zooplankton and invertebrate predators that would also shape the algal structure and relative abundance of each taxa. In any case, MALDI-TOF analysis of mature biofilms cultured in this study did not evidence bioactive compounds. Occasionally, loosely attached filamentous phototrophs, streamers, were observed floating in flow direction in biofilms at the mature phase. These structures could reflect an increase in surface area and a reduction in surface-boundary layer and secondly could be a mode of dispersal as these small communities could have better chance of survival when inhabiting a new environment (Figure 11a,b). 

This study contributed to describing the fate of an *O.* cf. *ovata* BHAB on artificial substrata under microcosm simplified, stable environmental conditions. It is often difficult to investigate biofilms in nature because of the large range of biotic and abiotic interactions that occur, the consequent ambiguity of experimental conditions, and the impossibility to maintain biofilm integrity during sampling procedures. Results indicated that at the low disturbance conditions tested, life-form strategies, competitiveness for resources, and possibly allelopathic interactions shaped the structure of the cultures during the biofilm growth. Toxin production did not occur in culture, although toxins were recorded in the inocula used for the experiments (0.137, 0.049, and 0.044 pg cell^−1^ of ovatoxin a in Run 1, Run 2, and Run 3, respectively). Phototrophs with a filamentous growth habit and surface-associated motility, such as tube-dwelling diatoms and cyanobacteria (thin, light orientated, trichomes), out-competed flagellated life-forms. Exploitation of more favourable compartments was obvious in development of streamers floating in the flow direction. These accounted for an increase in surface area and could be a favourable mode of dispersal. In the hydrodynamic conditions tested, *O.* cf. *ovata*, possibly relying on vertical migrations to reduce surface-boundary layers and resource limitation, was limited in its vegetative growth.

## Figures and Tables

**Table 1:** Physical environment on sampling; host organism type and fresh weight (FW).

Parameters	Site 1	Site 2
**Temperature (°C)**	27.7	27.0
**O_2_ (%)**	91.0	101.5
**Salinity (‰)**	30.0	37.0
**pH**	7.8	8.2
**Host substrata and FW**	4 bryozoan colonies (28.3 g Run 1, 72.8 g Run 2)	2 *Cystoseira* sp. thalli (95 g Run 3)
